# A four-compartment PBPK heart model accounting for cardiac metabolism - model development and application

**DOI:** 10.1038/srep39494

**Published:** 2017-01-04

**Authors:** Zofia Tylutki, Sebastian Polak

**Affiliations:** 1 Unit of Pharmacoepidemiology and Pharmacoeconomics, Department of Social Pharmacy, Faculty of Pharmacy, Jagiellonian University Medical College, Medyczna 9 Str., 30-688 Cracow, Poland; 2 Simcyp (a Certara Company) Limited, Blades Enterprise Centre, John Street, Sheffield S2 4SU, UK

## Abstract

In the field of cardiac drug efficacy and safety assessment, information on drug concentration in heart tissue is desirable. Because measuring drug concentrations in human cardiac tissue is challenging in healthy volunteers, mathematical models are used to cope with such limitations. With a goal of predicting drug concentration in cardiac tissue, we have developed a whole-body PBPK model consisting of seventeen perfusion-limited compartments. The proposed PBPK heart model consisted of four compartments: the epicardium, midmyocardium, endocardium, and pericardial fluid, and accounted for cardiac metabolism using CYP450. The model was written in R. The plasma:tissues partition coefficients (Kp) were calculated in Simcyp Simulator. The model was fitted to the concentrations of amitriptyline in plasma and the heart. The estimated parameters were as follows: 0.80 for the absorption rate [h^−1^], 52.6 for Kp_rest_, 0.01 for the blood flow through the pericardial fluid [L/h], and 0.78 for the P-parameter describing the diffusion between the pericardial fluid and epicardium [L/h]. The total cardiac clearance of amitriptyline was calculated as 0.316 L/h. Although the model needs further improvement, the results support its feasibility, and it is a first attempt to provide an active drug concentration in various locations within heart tissue using a PBPK approach.

According to current cardiac safety assessment guidelines, the focus is placed on *in vitro* hERG channel blockade^1^ as a predictive factor of *in vivo* QT interval prolongation^1^, which serves as a surrogate for torsades de pointes arrhythmia (TdP). However, the paradigm is shifting to other approaches. Among new recommended strategies are *in silico* mechanistic models of the human ventricular action potential and the assessment of drug effects on multiple cardiac currents in *in vitro* human models^2^,^3^.

The effect (extent of the delayed ventricular repolarization) should be related to the concentration of the test substance. The plasma concentration is most commonly used as the effective concentration surrogate due to the relatively high availability, yet it can be an imperfect substitute, especially for some types of chemical moieties (i.e., highly lipophilic). Drug concentration in heart tissue should be of particular interest regarding all possible sites where the drug might meet cardiac ion channels. It should gain even more prominence in light of reports that note that myocardial drug concentration better correlates with a change in QT length^4^,^5^ and that the tissue drug concentration profiles do not necessarily correlate with those in plasma^6^. Although measurements of drug concentration in human cardiac tissue seem impossible in patients not undergoing open heart surgery, the mathematical models do not face such limitations. A physiologically based pharmacokinetic (PBPK) modeling approach is considered a useful tool in tracking the concentration-time profiles of drugs in different tissues, based on *in vitro* data. Moreover, the mechanistic nature of PBPK allows for the creation of universal, drug-independent models^7^,^8^. The power of the idea lies in the fact that parameters describing PBPK models have their physiological meaning and that the compartments represent body’s organs and tissues. In recent years, there has been a significant rise in the interest of PBPK modeling by the pharmaceutical industry, impacting regulatory decision making. The models not only predict the pharmacokinetics but also can predict local drug concentrations to assess drug efficacy and safety^7^,^9^,^10^. A whole-body PBPK model usually treats tissues as homogeneous, ‘well-stirred’ compartments, not accounting for regional differences in drug concentrations within the tissue. Thus, Gaohua *et al*.^11^ proposed a multicompartment permeability-limited lung PBPK model applied to predict tuberculosis drug levels in the epithelial lining fluid and the lung tissue mass. Westerhout *et al*.^12^ developed a system-based pharmacokinetic model to describe the intra-brain drug distribution in rats. Neuhoff *et al*.^13^ built a PBPK kidney model under the name ‘MechKim’ to predict renal elimination. The model was also capable of simulating drug-drug interactions at the level of transporter inhibition^14^. According to our knowledge, there is a lack of such a detailed PBPK model for heart tissue. We aimed to fill the gap by providing a four-compartmental heart PBPK model structure as a basis for predicting drug distribution within cardiac tissue.

## Materials and Methods

### System-related input parameters

#### Whole-body PBPK model input parameters

The basic whole-body PBPK after Jones and Rowland-Yeo^8^ was implemented and used as a scaffold in which to nest the developed multicompartment heart model. The model assumed perfusion rate-limited kinetics, which means that the blood flow to the tissues (compartments) was the limiting process. The values of body weight-dependent fractional tissue volumes, fractional tissue blood flows, cardiac output and microsomal protein per gram of human liver (MPPGL) were from the provided code [Table 1]. The model assumed a reference human of 70 kg.

The perfusion rate-limited model works under the assumption that total drug concentrations in the tissue and in the plasma at steady state are in the equilibrium with each other. Tissue-to-plasma water partition coefficients (Kps), which define that concentration balance, were predicted in Simcyp Simulator v.14.1 using the Rodgers *et al*. method^15^,^16^. Kp defining the drug partition between plasma and the rest of the body (Kp_re_) was fitted. Non-linear hepatic metabolism according to Michaelis-Menten enzyme kinetics was incorporated into the model equations. The values of the maximal rate of saturating substrate concentrations (*V*_max_) in [mg/h] for each CYPs isoform considered in the model were calculated according to the Equations (1).


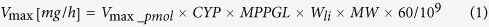


where:

*V*_max_*pmol*_-maximal rate of saturating substrate concentrations [pmol/min/pmol of *CYP*], *CYP-CYP* abundance in average human liver [pmol/mg of microsomal protein],

*MPPGL* - Microsomal protein per gram of human liver [mg/g]

*V*_*li*_ - total liver volume [L]. *MW*-molecular weight of the compound [g/mol],

#### Heart model input parameters

The physiological and anatomical data were derived from the literature to reflect the physiology of the normal human heart. They are presented in Table 2.

The parameter describing blood flow through the pericardium (Q_pf_) was unknown and fitted in the optimization process. It was subtracted from the Q_he_ to keep the sum of blood flow rates equal to cardiac output specified in the model. The Kps values between plasma and the epicardium (Kp_epi_), midmyocardium (Kp_mid_), and endocardium (Kp_endo_) were in the same ratio as those derived from Garcia *et al*.’s publication on saxitoxin poisoning^17^, i.e., 1:2.5:5, respectively. We took into account drug elimination in cardiac tissue as cytochrome P450 enzymes were detected in human cardiovascular tissue^18^,^19^,^20^. The values of mean microsomal fractions of the human heart for CYP enzymes (CYP2C8, CYP2C9, and CYP2J2) were used in computing total cardiac clearance in Equations (2) and (3). Heart muscle density was assumed to equal 1.0 g/mL^21^,^22^.


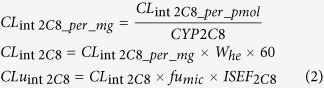


where:

*CL*_int2*C*8_*per_mg*_ - intrinsic clearance per mg of protein [mcL/min/mg],

*CL*_int2*C*8_*per_pmol*_ - intrinsic clearance per pmol of CYP2C8 isoform [mcL/min/pmol],

*CYP*2*C*8 - Mean CYP2C8 enzyme abundance in the human heart [pmol/mg],

*CL*_int 2*C*8_ - Intrinsic clearance for CYP2C8 in the human heart [L/h],

*W*_*he*_ - average heart weight [g],

*CLu*_int 2*C*8_ - intrinsic clearance for CYP2C8 based on unbound fraction of compound [L/h],

*fu*_*mic*_ - fraction of drug unbound in an *in vitro* microsomal preparation,

*ISEF*_2*C*8_ - Inter System Extrapolation Factor for CYP2C8.

Intrinsic clearances based on unbound fractions of amitriptyline for CYPs 2C9 (CLu_int2C9_) and 2J2 (CLu_int2J2_) were calculated analogically and added to calculate the total heart metabolic clearance according to Equation (3).


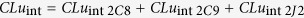



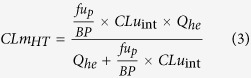


where:

*CLu*_int_ - total unbound intrinsic clearance per heart [L/h],

*CLm*_HT_ - total heart metabolic clearance [L/h],

*Q*_*he*_ - total heart blood flow [L/h],

*fu*_*p*_ - fraction of drug unbound in plasma,

*BP* - blood to plasma concentration ratio.

#### Drug-specific input parameters

The proposed PBPK model incorporating the permeability-limited (assuming the permeability across cell membranes being the limiting process) heart model was used to simulate plasma and cardiac concentrations of amitriptyline as an exemplary drug^23^. Physicochemical and PK parameters for amitriptyline used in a simulation scenario are listed in Table 3.

The oral bioavailability of amitriptyline is highly variable, ranging from 33 to 62%; thus, the fraction absorbed (F_abs_) was fixed at 0.5 (50%)^24^,^25^. The value of the first order absorption rate (k_a_) could not be found in reports, so k_a_ [h^−1^] was adjusted to the observed data. The percentage of unbound drug in the plasma (fu_p_) differs between studies from 3% to 7.4%^24^,^25^,^26^,^27^,^28^,^29^,^30^, so the fu_p_ value was fixed at 0.05 (5% of the free fraction). Because of the lack of information regarding the amitriptyline free fraction in pericardial fluid (fu_pf_), the fu_pf_ was assumed to equal fu_p_. A blood to plasma ratio (BP) of 1.04 was derived from^25^. The utilized value is in the range of other measured values, i.e., 0.86–1.13^24^,^31^.

The fixed Kp values were 3.0 (Kp_epi_), 7.4 (Kp_mid_)^32^, and 14.0 (Kp_endo_). The Kp value between plasma and pericardial fluid was fixed at 2.6^33^.

The liver metabolism considered amitriptyline biotransformation by CYP isoforms 1A2, 2B6, 2C8, 2C9, 2C19, 2D6, and 3A4^34^,^35^. The values of the maximal rate of saturating substrate concentrations (Vmax), Michaelis constants (Km) and Inter System Extapolation Factors (ISEF) used in the model are reported in the Table (4). We used 0.014 as the value of unbound fraction in hepatocytes (fu_h_), which was reported to be the highest bound of the range of fuh studied for imipramine and propranolol by Hallifax and Houston^36^. The intrinsic clearances denoting amitriptyline N-demethylation by CYPs 2C8 and 2C9 were 0.072 and 0.079 mcL/min/pmol^37^. Amitriptyline does not undergo CYP 2J2 metabolism^38^. fu_mic_ and ISEF factors were assumed to equal 1. The literature value of octanol/water partition coefficient (Log P) was 4.62^39^.

#### Clinical and simulation data

The mean plasma concentrations of amitriptyline after administration of a single oral dose of 25 mg (as amitriptyline is taken as a hydrochloride salt, pure amitriptyline counts for 22 mg to which the model was fitted) were observed by Curry *et al*.^40^ in ten healthy men. The corresponding maximal concentration in heart tissue was set to 114.35 ng/mL at 3.5 h postdose based on pig data^41^. Two additional concentration values in heart tissue referring to the absorption phase and two in the elimination phase were set to make the fitting process feasible.

#### Software used

The model was written in R v.3.3.0. Numerical solutions were computed using deSolve library v.1.13^42^. The system of ordinary differential equations was integrated using the LSODA method^43^. Model fitting was performed with algorithms from FME package v.1.3.2^44^. The weighted residuals (res_i,l_) of the model output versus any observed data point *i,* of observed variable *l*, were estimated by *modCost* function according to the Equation (4).


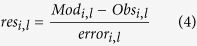


where Mod_i,l_ is the modeled value, Obs_i,l_ is the observed value, and error_i,l_ is a weighting factor. For error_i,l_ the *modCost* default value, i.e. 1, was chosen.

**Figure 1:**
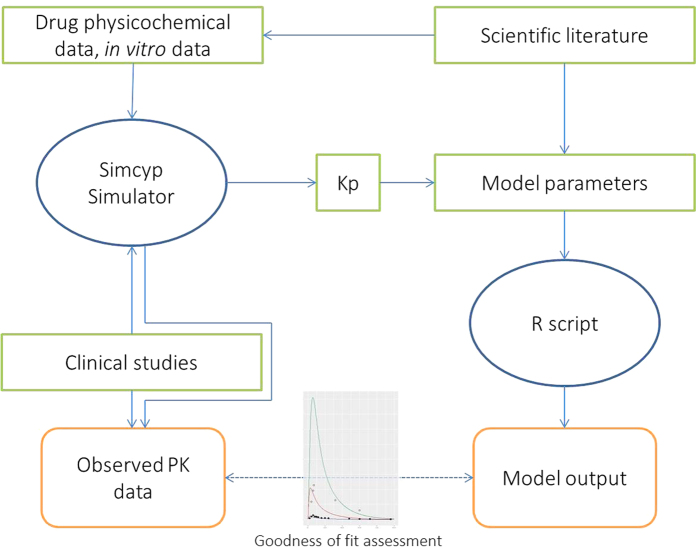
The modeling workflow. The color codes are as follow: blue, computational tool; green, data sources; orange, final outputs.

Simcyp Simulator^45^,^46^ v 14.1 (a Certara company) was used to predict Kp values for tissues represented in the full PBPK model. The diagram (Fig. 1) depicts the modeling workflow.

## Results

### Model structure

**Figure 2:**
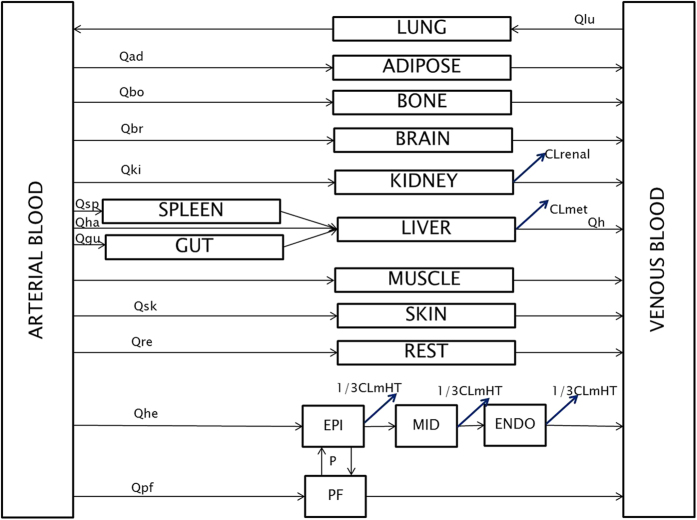
The structure of the final whole-body PBPK model. The model consisted of seventeen perfusion-limited compartments. The heart tissue was represented by four compartments, i.e., epicardium (EPI), midmyocardium (MID), endocardium (ENDO), and pericardial fluid (PF).

The structure of the final whole-body PBPK model is shown in Fig. 2.

The following is written as a set of ordinary differential equations (Equation 5 in the Appendix).

The model consisted of seventeen perfusion-limited compartments representing the following tissues: arterial blood, lung, adipose, bone, brain, kidney, spleen, gut, liver, muscle, skin, the rest of the body, venous blood, and the heart. Four compartments representing heart tissue were embedded into the whole-body PBPK structure. The added compartments stood for epicardium, midmyocardium, endocardium, and pericardial fluid, defined by the following volumes [L]: 0.0329, 0.0987, 0.1974, and 0.03, respectively. Epicardial, midmyocardial, and endocardial cells were set to constitute 10%, 30%, and 60% of the total heart tissue mass, according to Drouin *et al*.^47^.

The blood flow to the epicardium, midmyocardium, and endocardium was assumed to be equal, i.e., 4% of the cardiac output^8^. Arterial blood was assumed to perfuse the heart wall from the outward layer (epicardium), through the midmyocardium, to the most inner layer (endocardium). The blood left the cardiac tissue from the endocardium and returned to the venous blood compartment.

The pericardial fluid was perfused directly by arterial blood. Blood leaving the pericardial fluid entered the venous blood compartment directly.

The model also accounted for a drug disposition between the cardiac mass and pericardial fluid; it was described by passive disposition through the epicardial layer occurring in both directions. The parameters describing the permeability-limited process were grouped into one parameter (P) to simplify the model.

Physiological parameters describing the model structure were assumed to be constant, the compartments were homogeneous, and the equilibrium between the blood and tissues was reached immediately. The values of fu_p_ and fu_pf_ were assumed to be equal.

The proposed model assumed that the elimination occurred from the liver, kidney, and heart (tissue mass compartments). The implemented elimination clearances from the epicardium, midmyocardium, and endocardium contributed one-third each of the total cardiac metabolic clearance.

### Modeling the PK of amitriptyline

The Kps predicted in Simcyp Simulator for the amitriptyline compound are presented in Table 5.

The calculated amitriptyline clearances was 0.316 L/h for total cardiac clearance. The renal clearance was set as 0.504 L/h according to Turner *et al*.^37^.

The first simulation was run to fit Kp_re_ and k_a_ [h^−1^] parameters to the observed amitriptyline concentration in plasma by Curry *et al*.^40^. Q_pf_ [L/h] and P [L/h] were fixed at 0.01 and 0.40 as start parameters. The estimates (SD - standard error, p-value) are: k_a_ = 0.80075 (0.03171, p = 4.33×10^−11^), and Kp_re_ = 52.60950 (7.64768, p = 2.66×10^-5^). These values were used in the second simulation performed to estimate Q_pf_ and P (the model was fitted to the set heart concentrations). The estimates (SD, p-value) are as follows: Q_pf_ = 0.01193 (0.09783, p = 0.911), P = 0.78230 (5.76931, p = 0.901) (Table 6).

**Figure 3:**
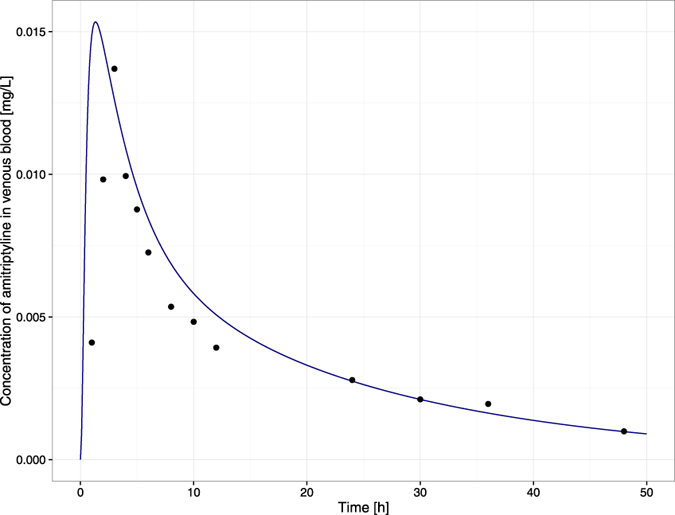
Simulation results. The time-amount of the amitriptyline profile (solid blue curve) resulted from fitting the model to values measured (solid black dots) by Curry *et al*.^40^.

**Figure 4:**
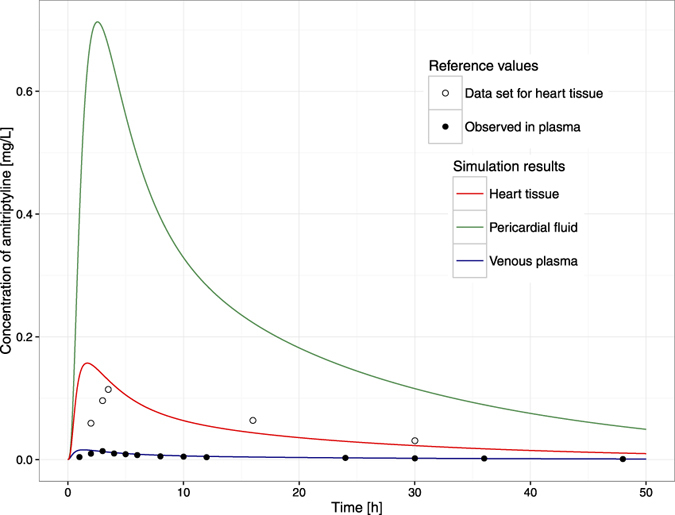
Simulation results. Time-concentration profiles of amitriptyline in venous plasma (solid blue curve), pericardial fluid (solid green curve), and heart tissue in total (solid red curve) resulted from fitting the model to values measured by Curry *et al*. in healthy volunteers’ plasma^40^ (solid black dots) and to data set according to the amitriptyline concentration observed in pig heart^41^ (open black circles).

**Figure 5:**
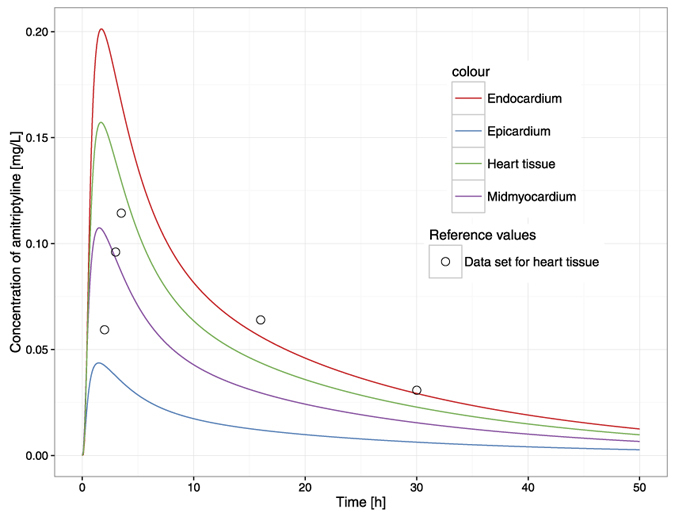
Simulation results. Time-concentration profiles of amitriptyline in heart tissue in total (solid green curve), in epicardium (solid blue curve), midmyocardium (solid violet curve), and the endocardium (solid red curve) data set according to the amitriptyline concentration observed in pig heart^41^ is depicted as open black circles.

Simulated time-concentration profiles and observed/set data are presented in Figs 3, 4 and 5.

**Figure 6:**
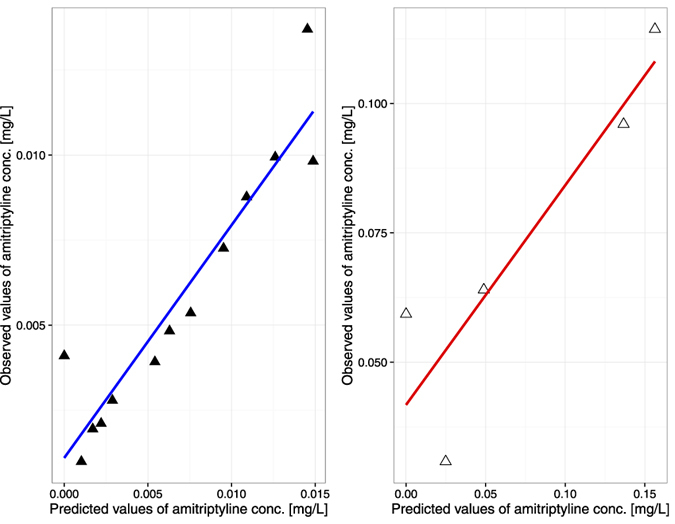
Observed values versus predicted values in the whole-body PBPK model. Solid black triangles represent data on the venous plasma amitriptyline amount. Open black triangles represent data on the amitriptyline amount in heart tissue.

**Figure 7:**
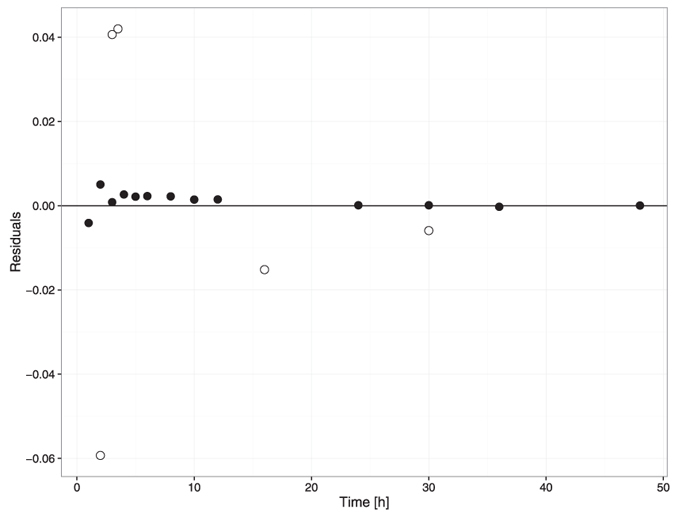
Plot of the residuals. Solid black dots refer to results from the 1^st^ simulation run to fit the model to the observed concentration in plasma^40^ (fitting Kp_re_ and k_a_ parameters). Open black dots refer to results from the 2^nd^ simulation run to fit the model to the data set on cardiac concentrations^41^ (fitting Q_pf_ and P parameters).

The goodness of fit is depicted in Figs 6 and 7. There was good agreement between the observed data and model-predicted profiles. However, the highest observed value was not captured. The plots revealed no systemic bias.

## Discussion

Regarding cardiovascular drug safety assessment and cardiac medication development, understanding active-site drug concentration is of great need. However, direct measurements pose problems, and prediction remains challenging. Because PBPK modeling makes predicting tissue concentration-time profiles possible, we focused on cardiac tissue and applied a PBPK quantitative mechanistic framework to develop a heart model structure.

Herein, the proposed heart model reflects a simplification of human cardiac anatomy and physiology. The assumption about equal blood perfusion throughout all three layers of the cardiac wall may be justified as it is claimed that “flow per gram of subendocardial myocardium is at least equal to flow per gram of subendocardial myocardium”^48^. The order of perfused compartments describes, in the simplest manner, the coronary tree that, arising from the right and left coronary arteries, forms a subepicardial system whose branches perforate more inner heart wall layers^49^. Pericardial fluid hypothetically forms in two ways, i.e., first by plasma ultrafiltration and secondly by the overflow of a small amount of interstitial fluid from the underlying myocardium^50^,^51^. Thus, blood flow and diffusion from a neighboring epicardial layer were applied in the model as possible routes for drug distribution within cardiac tissue. The estimate of Q_pf_ at 0.01 L/h, which contributes 0.076% of total cardiac blood flow, seems realistic although there are no published measured results to support that value.

Amitriptyline was chosen as a subject of the simulation because tricyclic antidepressants are said to distribute within tissues extensively^32^,^52^ and to induce cardiotoxicity^23^. According to CredibleMeds classification^53^ supported by the published case reports of TdP^54^, amitriptyline poses a conditional risk of TdP arrhythmia. Thus, its concentration in cardiac tissue is worth being studied.

Regarding predicted amitriptyline absorption, the highest plasma concentration was achieved 1.3 h postdose whereas mean T_max_ in the Curry *et al*.^40^ publication was 2.5 h. These differences most likely result from the oversimplified equation describing the absorption process. The estimated absorption rate constant being 0.80 [h^−1^] refers to the absorption of amitriptyline according to the first order kinetics. The more complex solution will be proposed in the next step of developing the model structure described herein.

The perfusion-limited distribution followed the Kps values predicted in Simcyp Simulator (Table 5). Kp_re_ value defining the drug partition between plasma and the rest of the body was estimated as 52.6 suggesting a higher accumulation of amitriptyline in parts of the human body other than considered in our model, or the active transport being engaged in the disposition process. The model-predicted amitriptyline concentrations in the heart sub-model compartments, ranked in ascending order, are as follows: epicardium, midmyocardium, endocardium, and pericardial fluid. The maximal heart concentration is achieved at 1.7 h postdose. At that time point, the ratios of the concentrations in the sites in question to the concentration in venous blood are 2.89 for epicardium, 7.13 for midmyocardium, 13.41 for endocardium, 42.65 for pericardial fluid, and 10.47 for the total heart. All of these values (except for pericardial fluid, which is too high according to postmortem findings^34^ and pericardial fluid composition is similar to plasma^55^) are within the range of heart Kp values reported in the forensic and animal studies^41^,^56^,^57^, which supports the feasibility of the model and will be further tested to validate effective concentration surrogates. The disposition within the cardiac wall follows the assumed pattern after a postmortem study by Garcia *et al*.^17^; however, in animal studies, there were no appreciable gradients observed and homogeneous transmural drug distribution was described^58^,^59^. Therefore, investigating the disposition between cardiac mural layers requires more mechanistic insight, most likely with a permeability-limited kinetics assumption. The same case is with predicting drug concentration in the pericardial fluid compartment. Without the support of more mechanistic data allowing to describe the permeability process across the membrane of the epicardium, the P parameter, corresponding in fact to the clearance of interstitial fluid, was estimated to be 0.78 [L/h] leading to predict the concentration of amitriptyline in pericardial fluid higher than expected.

The model incorporates drug metabolism in cardiac tissue as some CYP450 enzymes are highly expressed in the heart^20^,^60^, and according to Michaud *et al*., “could be extremely relevant for the local clearance of drugs and metabolite formation in the heart.” Indeed, amitriptyline is metabolized to nortriptyline, which together with its metabolites (Z-10-hydroxy-nortriptyline, E-10-hydroxy-nortriptyline) can contribute to cardiotoxicity^61^. Therefore, its concentration in the heart should also be taken into account in assessing the cardiac effect of the parent drug. That will be done by expanding the current model with the sub-model for the metabolite.

In order to assess the drug triggered cardiac effect, *in vitro* – *in vivo* extrapolation approach will be utilized. The patient-specific parameters and the individual drug concentration simulated by the PBPK heart model will be combined with the *in vitro* measured ion channels inhibition. Translation to the human *in vivo* situation will be done with the use of the Cardiac Safety Simulator (CSS) and expressed as the pseudoECG signal^62^,^63^. The pharmacodynamic models built-in to CSS that can be used are O’Hara-Rudy^64^ and ten Tusscher^65^,^66^ models. The simulation results, namely parameters characterizing depolarization (QRS) and repolarization (QT), will be compared against the available clinical data taking into account intra- and inter-individual human variability^67^,^68^.

## Conclusion

We described the development of a four-compartmental heart model and its nesting into a whole-body PBPK model. The model integrated literature-derived data on cardiac anatomy and physiology and was used to predict amitriptyline concentration in venous plasma, epicardium, midmyocardium, endocardium, and pericardial fluid. Our PBPK heart sub-model requires further development, but it represents a first attempt to provide an active drug concentration in various locations within heart tissue with the use of a PBPK approach.

## Additional Information

**How to cite this article**: Tylutki, Z. and Polak, S. A four-compartment PBPK heart model accounting for cardiac metabolism - model development and application. *Sci. Rep.* **7**, 39494; doi: 10.1038/srep39494 (2017).

**Publisher's note:** Springer Nature remains neutral with regard to jurisdictional claims in published maps and institutional affiliations.

## Supplementary Material

Supplementary Information

## Figures and Tables

**Table 1:** Whole-body PBPK input parameters after Rowland and Yeo^8^.

Parameter	Value	Unit
Fractional adipose volume	0.213	L/kg
Fractional bone volume	0.085629	L/kg
Fractional brain volume	0.02	L/kg
Fractional gut volume	0.0171	L/kg
Fractional heart volume	0.0047	L/kg
Fractional kidney volume	0.0044	L/kg
Fractional liver volume	0.021	L/kg
Fractional lung volume	0.0076	L/kg
Fractional muscle volume	0.4	L/kg
Fractional skin volume	0.0371	L/kg
Fractional spleen volume	0.0026	L/kg
Fractional venous volume	0.0514	L/kg
Fractional arterial volume	0.0257	L/kg
Fractional plasma volume	0.0424	L/kg
Fractional erythrocytes volume	0.0347	L/kg
Fractional rest of body volume	0.109771	L/kg
Cardiac output	108.33	mL/s
Fractional adipose blood flow	0.05	—
Fractional bone blood flow	0.05	—
Fractional brain blood flow	0.12	—
Fractional gut blood flow	0.146462	—
Fractional heart blood flow	0.04	—
Fractional kidney blood flow	0.19	—
Fractional hepatic blood flow (venous side)	0.215385	—
Fractional lung blood flow	1	—
Fractional muscle blood flow	0.17	—
Fractional skin blood flow	0.05	—
Fractional spleen blood flow	0.017231	—
Fractional rest of body blood flow	0.114615	—
Microsomal Protein Per Gram of Human Liver (MPPGL)	45	mg/g

**Table 2:** Physiological and anatomical data on heart tissue used as heart PBPK model-specific input parameters.

Parameter	Symbol	Value	Unit	References
Fraction of total heart volume for epicardium	V_epi_/V_he_	0.1	—	47
Fraction of total heart volume for midmyocardium	V_mid_/V_he_	0.3	—	47
Fraction of total heart volume for endocardium	V_endo_/V_he_	0.6	—	47
Volume of pericardial fluid	V_pf_	0.03	L	47
Relationship between Kps for heart layers	Kp_epi_:Kp_mid_:Kp_endo_	1:2.5:5	—	17
Mean CYP2C8 enzyme abundance in human heart	CYP2C8	0.2	pmol/mg	19
Mean CYP2C9 enzyme abundance in human heart	CYP2C9	5.5	pmol/mg	19
Mean CYP2J2 enzyme abundance in human heart	CYP2J2	0.17	pmol/mg	19

**Table 3:** Amitriptyline input parameters used in the simulation.

Parameter	Symbol	Value	Unit	Reference
Fraction of administered dose absorbed	F_abs_	0.5	—	24,25
Fraction of unbound drug in plasma	fu_p_	0.05	—	24, 25, 26, 27, 28, 29, 30
Fraction of unbound drug in pericardial fluid	fu_pf_	0.05	—	Assumption
Blood to plasma concentration ratio	BP	1.04	—	25
Midmyocardium to plasma partition coefficient	Kp_mid_	7.4	—	32
Pericardial fluid to plasma partition coefficient	Kp_pf_	2.6	—	33
Fraction of unbound drug in hepatocytes	fu_h_	0.014	—	36
Renal clearance	CL_renal_	0.504	L/h	37
Intrinsic clearance per pmol of CYP2C8	CL_int2C8_per_mol_	0.072	mcL/min/pmol	34
Intrinsic clearance per pmol of CYP2C9	CL_int2C9_per_mol_	0.079	mcL/min/pmol	34
Intrinsic clearance per pmol of CYP2J2	CL_int2J2_per_mol_	0.000	mcL/min/pmol	38
Fraction of unbound drug in an *in vitro* microsomal preparation	fu_mic_	1	—	Assumption
Inter System Extrapolation Factor	ISEF	1	—	Assumption
Log of the octanol-water partition coefficient for the compound	Log P	4.62	—	39

**Table 4:** Values of parameters used to describe non-linear hepatic metabolism according to Michaelis-Menten enzyme kinetics.

	CYP1A2	CYP2C9	CYP2C19	CYP2D6	CYP3A4	CYP2C8	CYP2B6
V_max_ [pmol/min/pmolCYP]	1.79	3.97	4.22	1.49	3.37†	0.7	0.25
K_m_ [μM]	63.5	50.5	8.52	7.12	213.8	9.74	56.7
ISEF	11.1	5.73	3.07	0.74	3.92	3.7	3.7

Values are after^34^.

^†^Denotes another source of data i.e.,^35^.

ISEF values correspond to B-Cell Lymphoma model and are taken from Simcyp Simulator.

**Table 5:** Kp values predicted in Simcyp Simulator for the amitriptyline compound.

Adipose	Bone	Brain	Gut	Kidney	Liver	Lung	Muscle	Skin	Spleen
Kp_ad_	Kp_bo_	Kp_br_	Kp_gu_	Kp_ki_	Kp_li_	Kp_lu_	Kp_mu_	Kp_sk_	Kp_sp_
4.27	4.28	2.78	11.97	6.52	20.16	2.09	10.04	5.73	11.24

**Table 6:** Results of the fitting process.

Parameter	Value	SD
k_a_	0.80075	0.03171
Kp_re_	52.60950	7.64768
Q_pf_	0.01193	0.09783
P	0.78230	5.76931
