# Supplementary material for: A four-compartment PBPK heart model accounting for cardiac metabolism - model development and application
Source: Sci Rep. 2017 Jan 4;7:39494. doi: 10.1038/srep39494 (PMC5209692; doi:10.1038/srep39494)
Supplement: Supplementary Information [file srep39494-s1.pdf]

A four-compartment PBPK heart model accounting for cardiac metabolism - model development and application

Zofia Tylutki*[1], Sebastian Polak[1,2]

Appendix

The final whole-body PBPK model is written as a set of differential equations:

Equation 5 Set of ordinary differential equations describing the final whole-body PBPK model,

$$Absorption = k_a \times D \times F_{abs}$$

$$Venous = Q_{pf} \times (\frac{C_{pf}}{Kp_{pf}} \times BP) + Q_{ad} \times (\frac{C_{ad}}{Kp_{ad}} \times BP) + Q_{bo} \times (\frac{C_{bo}}{Kp_{bo}} \times BP) + Q_{br} \times (\frac{C_{br}}{Kp_{br}} \times BP) +$$

$$Q_{he} \times (\frac{C_{endo}}{Kp_{endo}} \times BP) + Q_{ki} \times (\frac{C_{ki}}{Kp_{ki}} \times BP) + Q_h \times (\frac{C_{li}}{Kp_{li}} \times BP) + Q_{pf} \times (\frac{C_{pf}}{Kp_{pf}} \times BP) +$$

$$Q_{mu} \times (\frac{C_m}{Kp_{mu}} \times BP) + Q_{sk} \times (\frac{C_{sk}}{Kp_{sk}} \times BP) + Q_{re} \times (\frac{C_{re}}{Kp_{re}} \times BP)$$

$$\frac{dA_{ad}}{dt} = Q_{ad} \times (C_{ar} - \frac{C_{ad}}{Kp_{ad}} \times BP)$$

$$\frac{dA_{bo}}{dt} = Q_{bo} \times (C_{ar} - \frac{C_{bo}}{Kp_{bo}} \times BP)$$

$$\frac{dA_{br}}{dt} = Q_{br} \times (C_{ar} - \frac{C_{br}}{Kp_{br}} \times BP)$$

$$\frac{dA_{gu}}{dt} = Absorption + Q_{gu} \times (C_{ar} - \frac{C_{gu}}{Kp_{gu}} \times BP)$$

$$\frac{dA_{epi}}{dt} = Q_{he} \times (C_{ar} - \frac{C_{epi}}{Kp_{epi}} \times BP) - \frac{1}{3} \times CLm_{HT} \times C_{epi} \times fu_p - P \times (C_{epi} - fu_{pf} \times C_{pf})$$

$$\frac{dA_{mid}}{dt} = Q_{he} \times (\frac{C_{mid}}{Kp_{mid}} \times BP - \frac{C_{endo}}{Kp_{endo}} \times BP) - \frac{1}{3} \times CLm_{HT} \times C_{mid} \times fu_p$$

$$\frac{dA_{endo}}{dt} = Q_{he} \times (\frac{C_{mid}}{Kp_{mid}} \times BP - \frac{C_{endo}}{Kp_{endo}} \times BP) - \frac{1}{3} \times CLm_{HT} \times C_{mid} \times fu_p$$

$$\frac{dA_{pf}}{dt} = -P \times (fu_{pf} \times C_{pf} - C_{epi}) + Q_{pf} \times (C_{ar} - \frac{C_{pf}}{Kp_{pf}} \times BP)$$

$$\frac{dA_{ki}}{dt} = Q_{ki} \times (C_{ar} - \frac{C_{ki}}{Kp_{ki}} \times BP) - CL_{renal} \times C_{ki} \times fu_p$$

$$\frac{dA_{li}}{dt} = Q_{ha} \times C_{ar} + Q_{gu} \times (\frac{C_{gu}}{Kp_{gu}} \times BP) + Q_{sp} \times (\frac{C_{sp}}{Kp_{sp}} \times BP) - Q_h \times (\frac{C_{li}}{Kp_{li}} \times BP) - (\frac{C_{liverfree} \times C_{\max\_CYP1A2}}{Km_{CYP1A2} + C_{liverfree}} \times ISEF1A2)$$
$$- (\frac{C_{liverfree} \times C_{\max\_CYP2C9}}{Km_{CYP2C9} + C_{liverfree}} \times ISEF2C9) - (\frac{C_{liverfree} \times C_{\max\_CYP2C19}}{Km_{CYP2C19} + C_{liverfree}} \times ISEF2C19) - (\frac{C_{liverfree} \times C_{\max\_CYP2D6}}{Km_{CYP2D6} + C_{liverfree}} \times ISEF2D6)$$
$$- (\frac{C_{liverfree} \times C_{\max\_CYP3A4}}{Km_{CYP3A4} + C_{liverfree}} \times ISEF3A4) - (\frac{C_{liverfree} \times C_{\max\_CYP2B6}}{Km_{CYP2B6} + C_{liverfree}} \times ISEF2B6) - (\frac{C_{liverfree} \times C_{\max\_CYP2C8}}{Km_{CYP2C8} + C_{liverfree}} \times ISEF2C8)$$

$$\frac{dA_{lu}}{dt} = Q_{lu} \times C_{ve} - Q_{lu} \times (\frac{C_{lu}}{Kp_{lu}} \times BP)$$

$$\frac{dA_{mu}}{dt} = Q_{mu} \times (C_{ar} - \frac{C_{mu}}{Kp_{mu}} \times BP)$$

$$\frac{dA_{sk}}{dt} = Q_{sk} \times (C_{ar} - \frac{C_{sk}}{Kp_{sk}} \times BP)$$

$$\frac{dA_{sp}}{dt} = Q_{sp} \times (C_{ar} - \frac{C_{sp}}{Kp_{sp}} \times BP)$$

$$\frac{dA_{ve}}{dt} = Venous - Q_{lu} \times C_{ve}$$

$$\frac{dA_{ar}}{dt} = Q_{lu} \times (\frac{C_{lu}}{Kp_{lu}} \times BP) - Q_{lu} \times C_{ar}$$

$$\frac{dA_{re}}{dt} = Q_{re} \times (C_{ar} - \frac{C_{re}}{Kp_{re}} \times BP)$$

$$\frac{dD}{dt} = -Absorption$$

Where:

A is the drug amount [mg], *D* is a dose [mg], *BP* is the blood to plasma ratio, *Q* is tissue blood flow [L/h], *Kp* is the tissue to plasma partition coefficient, *V* is the tissue volume [L], and *C* is the drug concentration [mg/L] calculated as:

Equation 6

$$C_{tissue} = {}^{A_{tissue}}\!/\!{}_{V_{tissue}}$$

$C_{liverfree}$ is C_{liver} corrected for drug binding in hepatocytes ($C_{liverfree} = C_{liver} \times fu_h$)

The subscripts refer to the tissues as follows: *ad* – adipose, *bo* – bone, *br* – brain, *gu* – gut, *he* – heart, *endo* – endocardium, *mid* – midmyocardium, *epi* – epicardium, *pf* – pericardial fluid, *ki* – kidney, *li* – liver, *lu* – lung, *mu* – muscle, *sk* – skin, *sp* – spleen, *ve* – venous blood, *ar* – arterial blood, *re* – the rest of the body. Q_{ha} (equal to Q_h - Q_{gu} - Q_{sp}) refers to the blood flow from the artery compartment to the liver, and Q_h refers to the blood flow from the liver to the venous compartment. Subscripts beginning with *CYP* denote corresponding isoforms of cytochrome P-450.
